# Cryptococcal Meningitis in an Immunocompetent Patient with a Ventriculo-Pleural Shunt

**DOI:** 10.1155/2020/7601757

**Published:** 2020-04-14

**Authors:** Raynieri Fernandez, Jose Henao, Catherine Creticos

**Affiliations:** ^1^Department of Internal Medicine, Advocate Illinois Masonic Medical Center, Chicago, IL, USA; ^2^Infectious Disease Specialist, Advocate Illinois Masonic Medical Center, Chicago, IL, USA

## Abstract

Cryptococcal meningitis is the most common form of infection caused by Cryptococcus yeast species, followed by pulmonary infection. It is an opportunistic infection seen in patients with impaired cell immunity, most frequently in HIV patients and solid organ transplant recipients; however, it can occur in patients with no apparent immunodeficiency. We describe the case of *Cryptococcus neoformans* meningitis in an immunocompetent patient with aseptic cerebrospinal fluid analysis which highlights the heterogeneity of this disease.

## 1. Introduction

Cryptococcal meningitis (CM) is the most common form of infection caused by *Cryptococcus* yeast species, followed by pulmonary infection [1, 2]. It is often an opportunistic infection in patients with impaired cell-mediated immunity like those with HIV/AIDS, sarcoidosis, organ and stem cell transplant patients, and those taking immunosuppressive medications [3]. Over one million cryptococcal infections occur annually worldwide [4].

*Cryptococcus* species are encapsulated saprophytic yeast widely distributed in the environment. Its polysaccharide capsule is a major virulence factor that protects the organism from environmental destruction and downregulates the immune response of the host by adaptively modulating capsule structure [5].

The two main species causing most human diseases are *Cryptococcus neoformans* and *Cryptococcus gattii*. *C. neoformans* causes most infections, and its environmental reservoirs include bird droppings, soil, and decaying vegetation. On the other hand, *C. gattii* has been traditionally associated with mild illness in immunocompetent individuals [6, 7].

The most common symptoms of CM include headache, typically subacute in onset, and confusion, although infrequent neck stiffness can be present in about 20% of patients [3]. The symptoms may be more subtle than with bacterial meningitis. Consider CM in a patient with subacute onset neurologic findings such as headache, memory loss, or confusion especially if he has impaired cell-mediated immunity.

Lumbar puncture with cerebrospinal fluid (CSF) analysis is used to confirm the diagnosis, and it usually shows low white blood cell count with mononuclear predominance, elevated protein, and low glucose [8]. Around 25–30% have a normal CSF profile. There are different methods to detect Cryptococcus such as direct light microscopy after India ink stain, cryptococcal antigen (CrAg), and fungal culture. The role of imaging studies is almost exclusively to evaluate for complications (e.g., hydrocephalus and cryptococcomas) [3].

## 2. Case Presentation

The patient is a 66-year-old female with a past medical history significant for hypertension, seizure disorder, and hydrocephalus status-post ventricular-pleural (VP) shunt that came to the emergency department due to altered mental status. The patient had hydrocephalus due to cerebellar tumor with leptomeningeal metastases diagnosed by tissue sample 20 years ago, complicated with compressive hydrocephalus and ventriculomegaly that was only treated with a VP shunt, and there is no information of other treatment options. At baseline, the patient is bed-bound, alert, and oriented to person, time, and lives in a nursing home. She was brought due to fluctuation in her mental status, inattention, nonverbal response, and multiple febrile episodes with a maximum temperature of 101.4 F (38.5 C) of 2 days of duration. History was limited due to the inability of the patient to provide additional information. She was recently admitted to the hospital two months ago due to the concern of gastrointestinal bleeding with negative esophagoduodenoscopy and colonoscopy.

**Figure 1:**
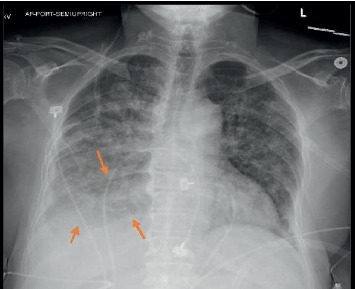
Mild to moderate cardiomegaly with atherosclerotic calcification along the aorta. Extensive patchy airspace opacities bilaterally. At least small sized layering right-sided pleural effusion (arrow). No detectable pneumothorax. Right-sided catheter/shunt along the chest wall, probably a ventriculo-pleural shunt.

**Figure 2:**
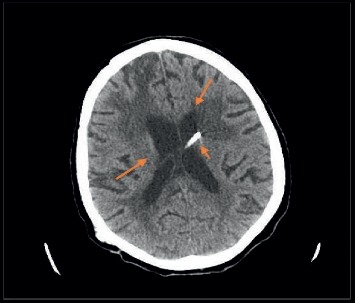
Right parietal approach ventriculostomy catheter with catheter tip terminating in or along the anterolateral aspect of the left lateral ventricle (short arrow). Interval re-expansion of the left lateral ventricle and left frontal horn prior to previous examination (long arrow). Postsurgical changes related to right frontal craniotomy and chronic microvascular ischemic.

Initial vital signs were significant for tachycardia 120 bpm, respiratory rate of 31  rpm, oxygen saturation 93% on venti-mask (9 L with FiO_2_ 40%), and blood pressure 140/70  mmHg. On physical examination, the patient was lethargic, only responsive to painful stimuli, decreased breath sounds on auscultation bilaterally with crackles on both lung bases. Initial laboratory workup was significant for white blood cell counts 20,800/mcl, lactic acid of 2.3, HIV nonreactive, and MRSA of the nares positive. Chest X-ray showed extensive patchy consolidation and ground-glass opacities throughout both lung fields with interstitial thickening more pronounced on the right lung base (Figure 1). Computed tomography (CT) of the head showed increased hydrocephalus compared to previous CT of the head compatible with shunt failure (Figure 2). Further investigation included ventricular-pleural shunt drainage and thoracentesis of the right-sided pleural effusion for fluid analysis. Initial CSF showed aseptic fluid (Table 1, #1 CSF 1) as well as aseptic pleural fluid. The patient was started on vancomycin and piperacillin-tazobactam for empiric coverage, and the initial diagnosis was altered mental status due to hospital-acquired pneumonia.

During the second day of hospital admission, meningitis/encephalitis polymerase chain reaction (PCR) panel was positive for *Cryptococcus neoformans* (Table 2, CSF 1). Induction therapy with amphotericin B and flucytosine were started, and ventricular-peritoneal shunt fluid analysis was repeated in addition to the antigen titers for confirmation. CSF fluid was reported as aseptic (Table 1, #1 CSF 2) with the persistence of Cryptococcus in the PCR (Table 2, CSF 2) by the third day of hospitalization. Pleural fluid cultures were consistent with the presence of cryptococcal infection as well.

Infectious disease team was consulted, and antimicrobials were tailored to amphotericin B and flucytosine to cover for cryptococcal meningitis and recommendations were made to change the ventricular-pleural shunt. Cryptococcal Ag serum titers were elevated (1 : 20), and fungal cultures later confirmed the presence of *C. neoformans* only on the pleural fluid by day 5. Through her hospitalization, the patient improved significantly and came back to her usual baseline by day 6, and repeated CSF evaluation was consistent with negative results and repeat fungal cultures did not show growth.

Eventually, during the second week of hospitalization, after confirming the absence of Cryptococcus by fungal culture and PCR, VP shunt was replaced for a ventriculoperitoneal shunt with an external ventricular device with resolving ventriculomegaly and hydrocephalus. The patient completed a total of 4 weeks of amphotericin B and flucytosine followed by 8 weeks of fluconazole oral 200  mg.

The patient was transition to the nursing home with close follow up with neurosurgery and infectious disease team.

## 3. Discussion

We present the case of an immunocompetent patient with cryptococcal meningitis. This case is unusual because it involved life-threatening CM caused by *C. neoformans* usually seen in the immunocompromised host [9]; nonetheless, the disease can occur in individuals with no apparent immunodeficiency [10]. Patients who have no known predisposing factors and develop severe pulmonary or extrapulmonary cryptococcosis are 17% to 22% of overall non-HIV, nontransplant (NHNT) population. Although these patients seem to be a homogeneous group, they probably represent the congruence of subclinical innate or acquired immunodeficiencies. Complications are more severe in this group, including more likely permanent neurologic sequelae [11].

Key elements of the management of CM are immediate antifungal therapy and reversal of immunosuppression when possible. Infectious Diseases Society of America (IDSA) recommends that the antifungal regimen have three phases: induction, consolidation, and maintenance. The first-line regimen for NHNT patients with meningitis are as follows: induction with liposomal amphotericin 3-4  mg/kg/day IV plus flucytosine for at least 4 weeks, consolidation with fluconazole 400–800  mg/day orally for 8 weeks, and maintenance with fluconazole 200–400  mg/day orally for 6–12 months [1]. Rapid diagnosis and treatment are crucial to the prognosis [12, 13].

Intracranial hypertension (ICH) is a severe problem in patients with CM. ICH both with and without ventriculomegaly can lead to irreversible neurological complications and increase morbidity and mortality. If the initial treatment, even combined with appropriate antifungal therapy, still fails to control ICH, the ventriculoperitoneal shunt could be an effective intervention. For instance, it was seen that even CSF pressure and Cryptococcus antigen counts could be significantly decreased after VP shunt in a retrospective collection data from 2010–2016 [14].

Our patient had a VP shunt implanted almost 5 years ago. Besides the recent hospitalization for suspected gastrointestinal bleeding, we were unable to delineate risk factors associated with CM. Further evaluation was inconclusive for apparent underlying immunodeficiency, and the CSF analysis was not contributive to the typical pattern seen, although CSF and pleural fluid cultures were consistent with the presence of *Cryptococcus neoformans*.

## 4. Conclusion

This case represents an unusual presentation of life-threatening cryptococcal meningitis caused by *C. neoformans* that it is usually seen in the immunocompromised host. The heterogeneity of CSF findings makes the diagnosis challenging when there are not well-known risk factors, or the history is unable to obtain as in our case. The rapid recognition by PCR panel allows proper treatment options early during the disease which is crucial to improve prognosis and survival of these patients.

## Figures and Tables

**Table 1:** Cerebrospinal fluid (CSF) analysis.

	CSF #1	CSF #2
CSF volume	10	12
Xanthochromia	Absent	Absent
CSF glucose	62	69
CSF protein	20	19
CSF nucleated cell count	1	1
CSF red blood cell count	None	None

**Table 2:** Meningitis/Encephalitis panel by PCR.

Meningitis/Encephalitis PCR panel	CSF #1	CSF #2
Varicella zoster virus	Not detected	Not detected
HIV antigen/antibody screen	Not detected	Not detected
*Streptococcus pneumonia*	Not detected	Not detected
*Streptococcus agalactiae*	Not detected	Not detected
Cytomegalovirus	Not detected	Not detected
*Listeria monocytogenes*	Not detected	Not detected
*Human parechovirus*	Not detected	Not detected
*Enterovirus*	Not detected	Not detected
*Neisseria meningitidis*	Not detected	Not detected
*Escherichia coli K1*	Not detected	Not detected
*Haemophilus influenzae*	Not detected	Not detected
Herpes simplex virus 1	Not detected	Not detected
Herpes simplex virus 2	Not detected	Not detected
*Human herpesvirus 6*	Not detected	Not detected
*Cryptococcus neoformans*	Positive	Positive
